# Worse outcome in breast cancer with higher tumor-infiltrating FOXP3+ Tregs : a systematic review and meta-analysis

**DOI:** 10.1186/s12885-016-2732-0

**Published:** 2016-08-26

**Authors:** Jiafeng Shou, Zhigang Zhang, Yucheng Lai, Zhigang Chen, Jian Huang

**Affiliations:** 1 Cancer Institute (Key Laboratory of Cancer Prevention and Intervention, National Ministry of Education, Provincial Key Laboratory of Molecular Biology in MedicalSciences), The Second Affiliated Hospital, Zhejiang University School of Medicine, Hangzhou, 31009 China; 2 Department of Oncology, Second Affiliated Hospital, ZhejiangUniversity School of Medicine, Hangzhou, 310009 China

**Keywords:** Breast cancer, FOXP3, TIL, Prognosis

## Abstract

**Background:**

Forkhead box P3(FOXP3) is known as the optimum maker for regulatory T cells(Tregs), which are conventionally thought to induce immune tolerance to disturb the antitumor immunity. However, the research on the prognostic significance of tumor-infiltrating FOXP3+ Tregs in breast cancer is still limited and the results are controversial.

**Methods:**

We searched for studies in PubMed, EMBASE and Web of Science prior to January 2015. The correlation between FOXP3+ tumor-infiltrating lymphocytes(TILs) and breast cancer prognosis was analyzed. The meta-analysis was performed using STATA 11.0. Pooled hazard ratios (HRs) with 95 % confidence intervals (CIs) were used to estimate the degree of the association between FOXP3+ TILs and prognosis of breast cancers, while relative ratios (RRs) were used to evaluate the relationship between FOXP3+ TILs and clinicopathological features of breast cancers.

**Result:**

A total of 15 studies comprising 8666 breast cancer patients met the inclusion criteria. Our results showed that higher FOXP3+ TILs level was significantly associated with poor prognosis in terms of overall survival (OS) (pooled HR:1.60, 95 % CI:1.06–2.42; *P < 0.05*). We found that breast cancer with higher FOXP3+ TILs level was positively correlated with c-erbB-2 positive status (pooled RR:1.52, 95 % CI:1.32–1.75; *P < 0.05*), lymph node positive status(pooled RR:1.17, 95 % CI:1.04–1.32; *P < 0.05*) while there was a negative association with ER positive status(pooled RR:0.65, 95 % CI:0.56–0.76; *P < 0.05*) and PR positive status(pooled RR:0.66, 95 % CI:0.51–0.87; *P < 0.05*).

**Conclusion:**

The present results of meta-analysis showed that higher FOXP3+ TILs level in patients with breast cancer led to poor overall survival (OS) and was significantly associated with c-erbB-2 status, lymph node status, ER status and PR status. FOXP3+ TILs level is a promising prognostic factor in breast cancer.

**Electronic supplementary material:**

The online version of this article (doi:10.1186/s12885-016-2732-0) contains supplementary material, which is available to authorized users.

## Background

Breast cancer is one of the most common malignancies and expected to account for 29 % of all newly diagnosed cancers and 14 % of all cancer deaths in women worldwide. Though death rate for female breast cancer has decreased by 35 % from peak rate, it is still the leading cause of cancer death in women from aged 20 to 59 years [1]. In most cancers, including breast cancer [2–4], infiltrating of FOXP3+ regulatory TILs have been reported to be associated with worse clinical outcome. However, MJM Gooden et al. reported that FOXP3+ regulatory TILs were not linked to the overall survival in cancers [2].

FOXP3 is a forkhead box transcription factor containing a DNA-binding domain that suppresses the expression of target genes [3]. The regulatory T lymphocytes (Tregs), subpopulation of CD4(+) T lymphocytes, is an important obstacle in antitumor immunity by suppression of tumor antigen reactive T lymphocytes [4–6]. The transcription factor FOXP3, known as the most specific marker of Tregs [7–9], plays a crucial role in the development and function of Tregs,. FOXP3 is constitutively expressed in the nucleus of human Tregs [10, 11]. Several studies showed that higher FOXP3+ Tregs indicated poor prognosis [12–14] in patients with breast cancer while some revealed no direct association between them [15]. In short, studies to confirm the clinical significance of FOXP3+ TILs in breast cancer are still insufficient and the prognostic value still lacks assessment, especially in different molecule types of breast cancer. Therefore, we find it necessary to further assess the association between tumor-infiltrating FOXP3+ Tregs and prognosis of breast cancer by conducting a meta-analysis with a large sample size (*N* = 8666). The relationship between FOXP3+ TILs and several clinicopathological features of breast cancer was also evaluated.

## Methods

### Literature search strategy

We searched PubMed, EMBASE and Web of Science for relevant studies before January 2015, using the terms: “FOXP3 lymphocytes” or “FOXP3 regulatory T cell” or “FOXP3 TIL” or “FOXP3 tumor-infiltrating lymphocytes” and “breast cancer”. We searched the references of all retrieved publications and conference proceedings again to identify additional relevant studies.

### Selection criteria

The studies included in our meta-analysis should meet following criteria: [1] the study must be conducted on the human beings; [2] the study must assess the association between FOXP3+ TILs level and the prognosis of breast cancer; [3] The count of FOXP3+ TIL include either tumor bed or tumor peripheral lymphocytes [4] the study must contain sufficient published data to determine an estimate of hazard ratio(HR) and a 95 % confidence interval(95 % CI); [5] original research article must be published in English.

### Data extraction and quality assessment

Data were extracted from the eligible studies by two investigators independently. Discrepancies were resolved by consensus. The following information was abstracted from all included publications: author, year of publication, country of study, tumor type, median follow-up time, cut-offs for positive expression, number of TILs-low and TILs-high patients, number of patients with different clinicopathological features, outcome of analysis (including HRs and 95 % CIs).

Quality assessment was conducted for each of the included studies using the Newcastle-Ottawa quality assessment scale [16]. The score assessed eight items on methodology that were categorized into three dimensions, including selection, comparability and outcome. Interpretation of the scale is performed by awarding “stars”, for high-quality element. The studies with 6 scores or more were regarded as high-quality ones in the scale.

### Statistical analysis

Survival outcome data were synthesized using the HR and its 95 % CI to assess the impact of higher FOXP3+ TILs level on the overall survival (OS) and relapse free survival (RFS) of patients with breast cancer. Several included studies have provided HRs and their 95 % CIs, which we extracted directly from the papers. Otherwise, we calculated the HRs and 95 % CIs from the Kaplan-Meier survival curve using the software Engauge Digitizer version 4.1 (http://digitizer.sourceforge.net/) [17, 18]. The relative ratio (RR) was used to evaluate the association between FOXP3+ TILs and clinicopathological features of breast cancer (including tumor category(T), lymph node category(N), c-erbB-2, ER and PR status). Heterogeneity across studies was evaluated using a Chi-square-based Q statistical test [19], and the I^2^ value was used to quantify the heterogeneity [20]. The random-effect model (the DerSimonian and Lairdmethod) was used for meta-analysis [21]. Publication bias was assessed using Begg’s test [22]. In addition, sensitivity analysis was performed to examine the stability of the pooled results. The statistical analyses were conducted using STATA11.0. All P values were two-sided, and *P* < 0.05 was considered to be statistically significant.

## Results

### Study selection and characteristics

**Fig. 1:**
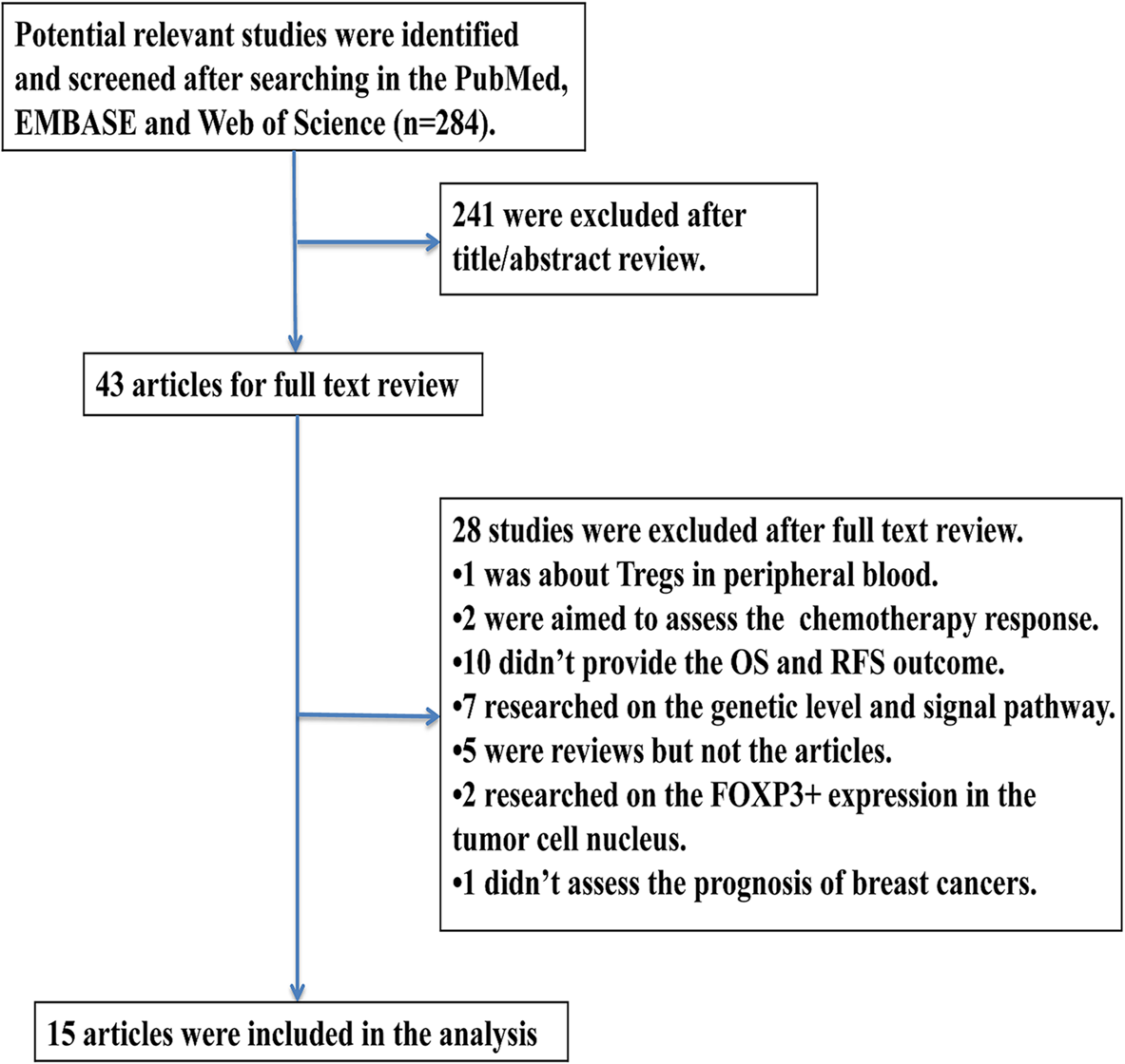
Flow diagram showing the study selection procedure

As shown in Fig. 1, a total of 284 articles were initially retrieved from the above databases using the search strategy described above. From candidate publications following exclusions, our search found 15 studies satisfying the inclusion criteria. The main characteristics of included studies were presented in Additional file 1: Table S1. An additional file shows this in more detail (see Additional file 1). These studies were published from 2006 to 2014 and total 8666 patients with breast cancer were enrolled. Individual sample size ranged from 72 to 3992 (mean 578). These studies were conducted in nine counties (the United Kingdom, the United States, Turkey, Korea, China, Canada, Japan, Australia and France). Overall, the included studies in meta-analysis were of high quality, six studies scored 8, six studies scored 7 and three studies scored 6.

### FOXP3+ TILs level and prognosis of breast cancers

**Fig. 2:**
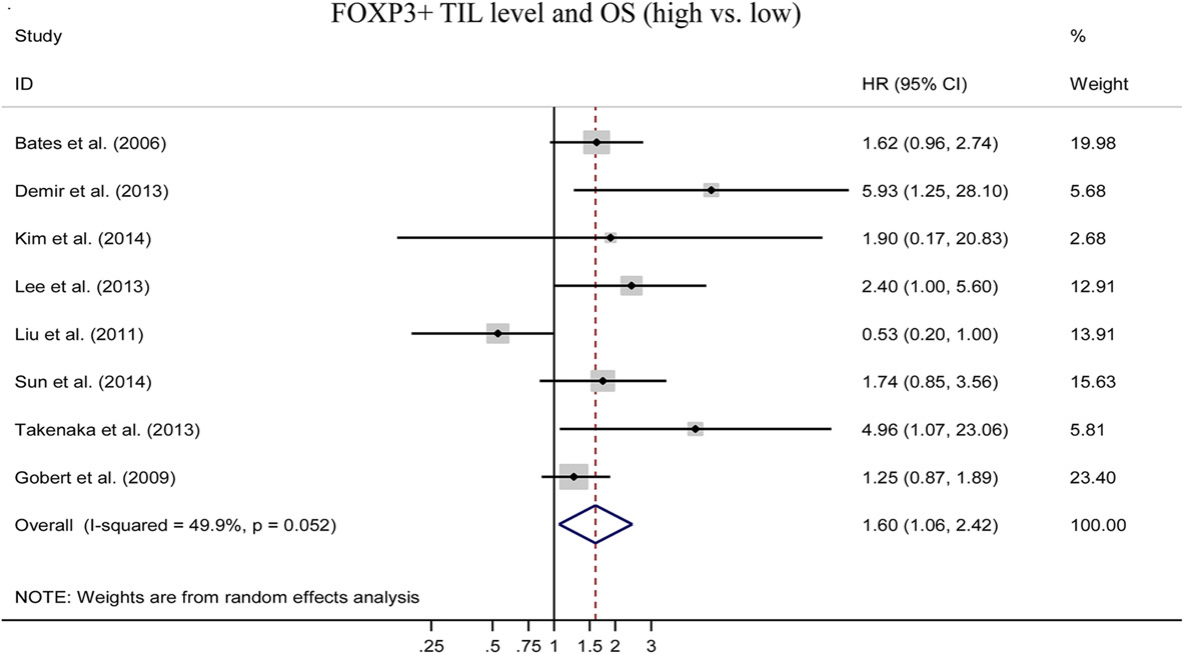
Forest plot of the hazard ratio (HR) for the association of FOXP3+ level with overall survival (OS) in breast cancer patients

**Fig. 3:**
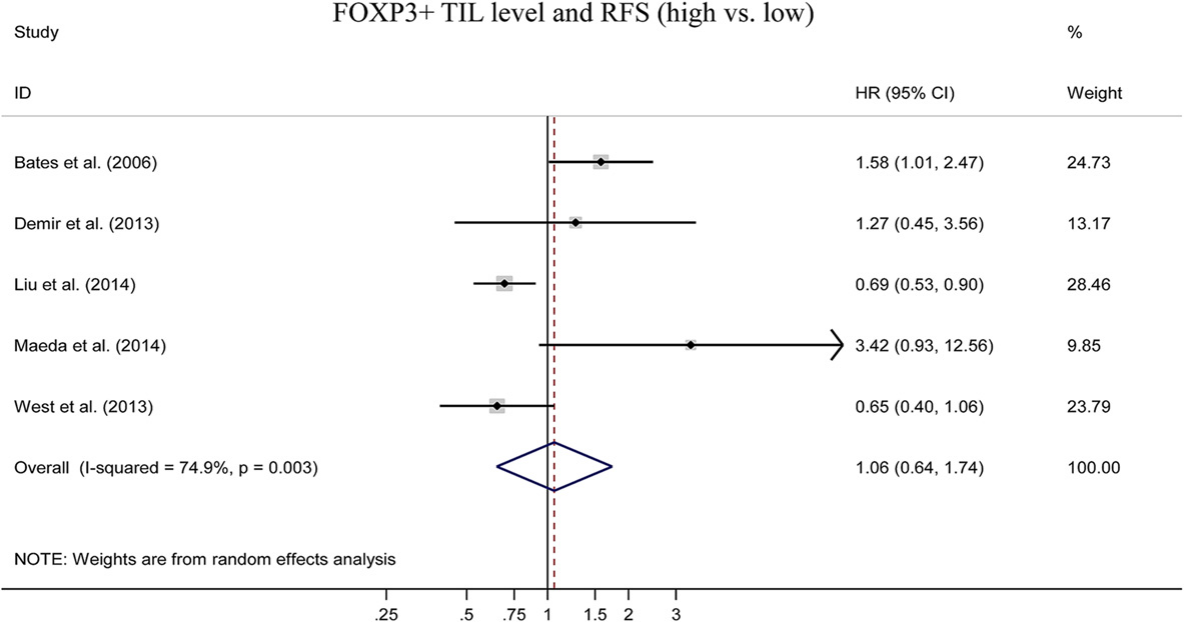
Forest plot of the hazard ratio (HR) for the association of FOXP3+ level with relapse free survival (RFS) in breast cancer patients

We pooled overall survival (OS) and relapse free survival (RFS) to assess the impact of FOXP3+ TILs level on the prognosis of breast cancers. 8 studies evaluated the relationship between FOXP3+ TILs and OS [12, 13, 23–28]. The pooled HR is 1.60(95 % CI:1.06–2.42; *P < 0.05*) (Fig. 2). It indicated that higher FOXP3+ TILs level was statistically related to a poor OS rate. 5 studies assessed the association between FOXP3+ TILs and RFS [14, 23, 28–30]. The pooled HR is 1.06(95 % CI: 0.64 ~ 1.74; *P > 0.05*) (Fig. 3) which indicated that FOXP3+ TILs level was not correlated with RFS of breast cancers.

### Correlation of FOXP3+ TILs level with clinicopathological parameters

**Fig. 4:**
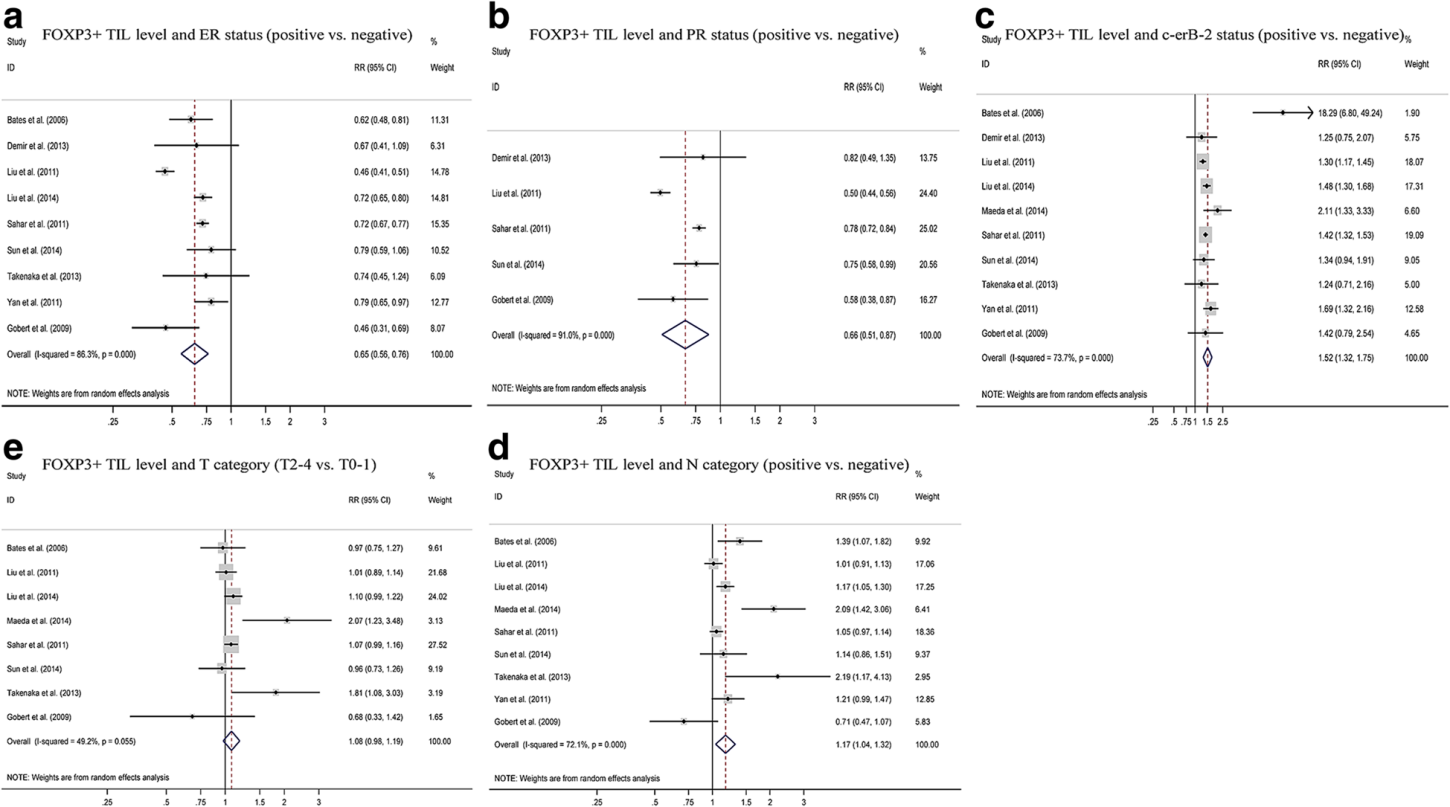
Forest plot assessing the FOXP3+ TILs and clinicopathological features such as ER status (**a**), PR status (**b**), c-erbB-2 status (**c**), tumor category (**d**), lymph node category (**e**)

The meta-analysis also estimated the association between FOXP3+ TILs level and certain clinicopathological parameters. As shown in Fig. 4c and e, breast cancers with higher FOXP3+ TIL level were positively correlated with c-erbB-2 status(pooled RR:1.52, 95 % CI:1.32–1.75; *P < 0.05*), lymph node status(pooled RR:1.17, 95 % CI:1.04–1.32; *P < 0.05*). Compared with ER-negative breast cancers, ER-positive breast cancers showed lower FOXP3+ TILs level (pooled RR:0.65, 95 % CI:0.56–0.76; *P < 0.05*) (Fig. 4a). Similar phenomenon was observed in PR-positive breast cancers(pooled RR:0.66, 95 % CI:0.51–0.87; *P < 0.05*) (Fig. 4b). This indicated that in c-erbB-2 positive or lymph node positive breast cancers, there was higher FOXP3+ TILs level. On the other hand, ER positive or PR positive breast cancers were accompanied with lower FOXP3+ TILs level. In addition, we found no association between higher FOXP3+ TIL level and tumor category of breast cancers (pooled RR:1.08, 95 % CI:0.98–1.19; *P > 0.05*) (Fig. 4d).

### Publication bias

**Fig. 5:**
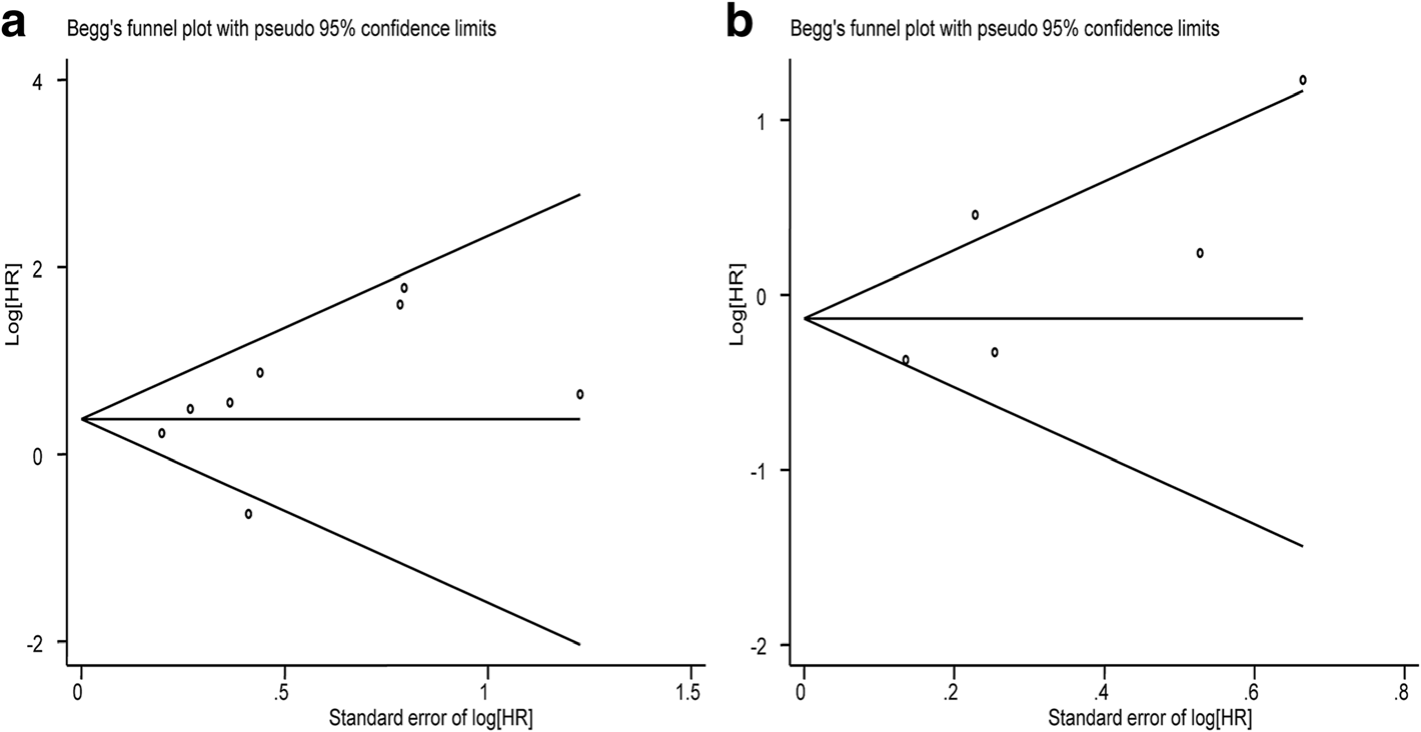
Begg’s test results of the OS (**a**) and RFS (**b**)

**Fig. 6:**
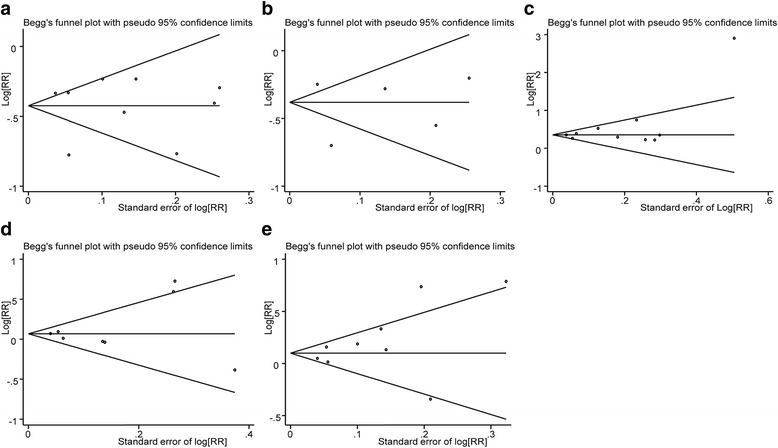
Begg’s test assessing the FOXP3+ TILs and clinicopathological features such as ER status (**a**), PR status (**b**), c-erbB-2 status (**c**), tumor category (**d**), lymph node category (**e**)

Begg’s test indicated negligible publication bias after assessing the funnel plot for the studies included in our meta-analysis (Figs. 5 and 6).

### Sensitivity analysis

In the sensitivity analysis, the influence of each study on the pooled HR of OS and RFS was assessed by repeating the meta-analysis while sequentially omitting each study. The results showed that the association did not change significantly after removing any study. An additional shows this in more detail (see Additional file 2).

## Discussion

FOXP3+ Tregs, part of tumor-infiltrating lymphocytes (TILs), play a critical role in immune tolerance and deficiency of anti-tumor immunity [4–6]. Several studies revealed that FOXP3+ TILs level had a negative impact on the prognosis of breast cancer [14, 31, 32]. However, the exact prognostic significance was still unclear and the information was limited. In our meta-analysis, 15 eligible articles were included which evaluated the association between FOXP3+ TILs level and prognosis of breast cancer. Ezzeldin M et al. and X. Yu et al. have reported that TILs were associated with prognosis of breast cancer in their meta-analysis [33, 34]. However, TILs in our meta-analysis referred to FOXP3+ TILs specifically. Thus, our meta-analysis seems more applicable in clinical practice. The pooled results showed that a higher density of FOXP3+ lymphocytes in tumor tissue was a promising prognostic factor for OS of breast cancers. However, the FOXP3+ TILs level was proved not having significantly higher risk of relapse. These results are potentially important for prognostic and treatment reasons.

FOXP3 is the most specific marker of CD4 + CD25+ Tregs and appears to be critical for the development and function of Tregs derived from thymus [8, 35, 36]. Tumor-infiltrating lymphocytes(TILs) in our meta-analysis refer to the lymphocytes either infiltrating in tumor bed or tumor periphery. FOXP3+ Tregs was an important group of TILs and Udaya K et al. has reported that FOXP3 + Tregs prevalence was 20.2 % in TILs of breast adenocarcinoma [37]. FOXP3+ Tregs suppressed the function of effector T cells to destroy maintenance of immune balance which resulted in the escape of tumor immunological surveillance [8, 38]. FOXP3+ Tregs secreted TGF-β, which indicated that the suppression of anti-tumor immunity of FOXP3+ Tregs may be cytokine-dependent [39]. FOXP3+ Tregs also secreted IL-10 to suppress Th1/2 cell proliferation via inhibition of IL-2 and down-regulating MHC class II on monocytes [40, 41]. In addition, FOXP3+ Tregs inhibit the proliferation and IFN-y secretion by activated CD8+ T cells and CD4+ helper T cells [37]. FOXP3+ Tregs express CD39/CD73 that generates adenosine, which can down-modulate immune function [42, 43]. These may account for the poor overall survival in breast cancers with higher FOXP3+ TILs level. Nevertheless, other immune cells might have impact on the prognosis of breast cancer. Natural killer (NK) cells contributed to a pathological complete response (pCR) in breast cancers following neoadjuvant chemotherapy [44]. Lymphocytes infiltrating human breast cancers showed lower levels of NK-cell activity [45]. Tiainen S et al. reported that higher level of infiltrated macrophage, especially M2- like, correlated with poor outcome in breast cancer [46]. Thus, a more effective model needs to be established to predict the prognosis of breast cancer. No association was observed between higher FOXP3+ TILs level and RFS of breast cancers in our meta-analysis.

In addition, we investigated the association between FOXP3+ TILs and some clinicopathological parameters of breast cancer. Significant positive correlations were found between high FOXP3+ TILs with c-erbB-2 status and lymph node status while negative correlations with ER and PR status. The result implied that ER+ breast cancer was accompanied with lower FOXP3+ TILs. N R West et al. [30] demonstrated a similar phenomenon with our result. Another point worth attention was that in c-erbB-2+ breast cancer, there was a significantly higher level of FOXP3+ TILs, also reported by Sahar M. A et al. [15].

However, there are still some limitations in our meta-analysis. Firstly, the included studies were restricted within English articles and completed in 9 countries, which may result in potential publication bias while Begg’s test showed no evidence. Secondly, heterogeneity was found in the meta-analysis, which may be due to different cutoff values of FOXP3+ TILs in the included studies. Though the detecting technology of FOXP3+ TILs in included studies was all immunohistochemistry (IHC), the different antibody type and antibody concentration may contribute to the heterogeneity of included studies. Thirdly, the HRs and 95 % CIs extracted from survival curves may be less reliable than those directly obtained from the articles.

## Conclusion

Despite the limitation listed above, our meta-analysis based on currently published articles indicted that higher FOXP3+ TILs level was inclined to poor OS while no significant association was observed with RFS. Accordingly, FOXP3+ TILs can be considered as a promising therapeutic target.

## Additional files

Additional file 1:
**Table S1.** Characteristics of the included studies. (DOCX 39 kb) Additional file 2: Sensitivity analysis of forest plots included in our meta-analysis. (PDF 43 kb)

## Abbreviations

FOXP3: Forkhead box P3
Tregs: Regulatory T cells
TILs: Tumor-infiltrating lymphocytes
HR: Hazard ratio
CI: Confidence interval
RR: Relative ratio
OS: Overall survival
RFS: Relapse free survival
T: Tumor category
N: Lymph node category
NK: Natural killer
pCR: Pathological complete response
IHC: Immunohistochemistry
