# Supplementary material for: Worse outcome in breast cancer with higher tumor-infiltrating FOXP3+ Tregs : a systematic review and meta-analysis
Source: BMC Cancer. 2016 Aug 26;16(1):687. doi: 10.1186/s12885-016-2732-0 (PMC5002190; doi:10.1186/s12885-016-2732-0)
Supplement: Additional file 1: — Table S1. Characteristics of the included studies. (DOCX 39 kb) [file 12885_2016_2732_MOESM1_ESM.docx]

| **Table S1 Characteristics of the included studies** | | | | | | | | | | | | | | | | |
| --- | --- | --- | --- | --- | --- | --- | --- | --- | --- | --- | --- | --- | --- | --- | --- | --- |
| **Author(year)** | **Country of study** | **Duration of**  **follow-up(range)** | **Type of cancer** | **Patient No.** | **Age, median**  **(range)** | **Stage** | **T** | **N(P/N)** | **M(M1/M0)** | **State of**  **c-erbB-2(P/N)** | **State of**  **ER(P/N)** | **State of**  **PR( P/N)** | **Technique for FOXP3+Treg assessment** | **Cut off value**  **(high/low)** | **Outcomes measured**  **HR(95%CI)** |  |
|  |  |  |  |  |  |  |  |  |  |  |  |  |  |  |  | **Study quality** |
| Bates et al.(2006) | United  Kingdom | 7.3year(0.2~11.3) | invasive breast cancer | 237 | NR | I(39);  II(62);  III(22) | High: T<=2cm(56); T>2cm(54)  Low: T<=2cm(54);  T>2cm(55) | High: 57/53  Low: 41/74 | NR | High: 17/84  Low: 6/95 | High: 60/50  Low: 88/27 | NR | IHC | ≥15/HPF(119/118) | OS: 1.62(0.96~2.74)  RFS：1.58(1.01~2.47) | 8 |
| Bohling et al.(2008) | America | NR | invasive breast carcinoma | 97 | NR | I(19);  II(26);  III(52) | NR | NR | NR | NR | NR | NR | IHC | ≥15/HPF | NR | 8 |
| Demir et al.(2013) | Turkey | 66.2months(34.9~97.5) | locally advanced breast cancer | 101 | NR | IIIA(19)  IIIB(76)  IIIC（6） | NR | NR | NR | High: 11/19  Low: 8/22 | High: 15/15  Low: 21/9 | High: 16/14  Low: 19/11 | IHC | ≥11.5(30/30) | OS: 5.93(1.25~28.1)  RFS: 1.27(0.45~3.56) | 6 |
| Kim et al.(2014) | Korea | 69months | invasive ductal carcinoma | 143 | NR | NR | NR | NR | NR | NR | NR | NR | IHC | NR | OS: 0.526(0.048~5.805)  DFS：3.513(0.967~12.766) | 6 |
| Kim et al.(2013) | Korea | 33.7months(21.9~38.3) | early breast cancer | 72 | 49(16-83) | I/II(59)  III(13) | NR | NR | NR | NR | NR | NR | IHC | ≥17/HPF | DFS: 0.00(0.00~1.631) | 7 |
| Lee et al.(2013) | Korea | 73.5months(24.2~120.0) | triple-negative breast cancer | 86 | NR | NR | NR | NR | NR | NR | NR | NR | IHC | ≥15/HPF（22/64） | OS: 2.40 (1.000~5.60) | 7 |
| Liu et al.(2011) | China | 66months(1~78) | invasive breast carcinoma | 1270 | 52(19-92) | I(86);  II(738);  III(323) | High: T<=2cm(167); T>2cm(479)  Low: T<=2cm(164);  T>2cm(460) | High: 371/27  Low: 354/270 | NR | High: 222/424  Low: 143/481 | High: 272/374  Low: 506/118 | High: 235/41  Low: 445/179 | IHC | ≥11/HPF(646/624) | OS: 0.53 (0.20~1.00) | 6 |
| Liu et al.(2014) | Canada | 12.6years(0.1~18.5) | breast cancer | 3992 | 58.9(23-95) | I(209);  II(1536);  III(2040) | High: T<=2cm(512); T>2cm(511)  Low: T<=2cm(1566);  T>2cm(1377) | High: 484/54  Low:1235/1720 | NR | High: 184/830  Low: 314/2486 | High: 641/388  Low: 2120/812 | NR | IHC | ≥2/core(646/624) | RFS: 0.69(0.53~0.90) | 7 |
| Maeda et al.(2014) | Japan | 67months(7.8~90.5) | invasive ductal carcinoma | 90 | NR | NR | High: T<=2cm(12); T>2cm(31)  Low: T<=2cm(28);  T>2cm(19) | High: 31/21  Low: 10/37 | NR | High: 27/16  Low: 13/34 | NR | NR | IHC | ≥9.7%(43/47) | RFS：3.416(0.929~12.564) | 7 |
| Sahar et al.(2011) | United  Kingdom | 128months(4~243) | invasive breast carcinomas | 1444 | NR | I(246);  II(474);  III(716) | High: T<=2cm(563); T>2cm(355)  Low: T<=2cm(341);  T>2cm(177) | High: 358/56  Low: 185/333 | High: 298/617  Low: 145/372 | High: 151/731  Low: 24/473 | High: 573/297  Low: 409/67 | High: 461/40  Low: 333/138 | IHC | ≥3/core(773/417) | NR | 8 |
| Sun et al.(2014) | China | 6years(0.67~8.52) | invasive breast cancer | 208 | 57.6(31-85) | I/II(177)  III(31) | High: T<=2cm(53); T>2cm(51)  Low: T<=2cm(51);  T>2cm(53) | High: 35/69  Low: 29/75 | NR | High: 13/91  Low: 7/97 | High: 76/28  Low: 85/19 | High: 59/45  Low: 73/31 | IHC | NR | OS: 1.74(0.85~3.56)  DFS：1.76(0.86~3.61) | 7 |
| Takenaka et al.(2013) | Japan | NR | invasive breast carcinoma | 100 | NR | I/II(77)  III(23) | High: T<=2cm(16); T>2cm(21)  Low: T<=2cm(42);  T>2cm(21) | High: 21/10  Low: 24/37 | NR | High: 10/27  Low: 13/50 | High: 18/19  Low: 38/25 | NR | IHC | score≥2+ | OS：4.96(1.07~23.06) | 8 |
| West et al.(2013) | Canada | 83months | ER- breast cancer | 144 | NR | NR | NR | NR | NR | NR | NR | NR | IHC | ≥18/mm2 | RFS: 0.647(0.397~1.056) | 8 |
| Yan et al.(2011) | Australia | 131.9months | breast cancer | 491 | 55(24-87) | I(81);  II(207);  III(195) | NR | High: 107/10  Low: 107/154 | NR | High: 20/186  Low: 7/238 | High: 135/81  Low: 189/73 | NR | IHC | ≥15/HPF | NR | 7 |
| Gobert et al.(2009) | France | 96months | breast cancer | 191 | NR | NR | High: T<=2cm(56); T>2cm(6)  Low: T<=2cm(109);  T>2cm(20) | High: 27/34  Low: 67/50 | NR | High: 7/54  Low: 8/110 | High: 42/18  Low: 114/13 | High: 38/22  Low: 103/25 | IHC | ≥18/HPF | OS: 1.25(0.87,~1.89) | 8 |
