# Supplementary material for: Worse outcome in breast cancer with higher tumor-infiltrating FOXP3+ Tregs : a systematic review and meta-analysis
Source: BMC Cancer. 2016 Aug 26;16(1):687. doi: 10.1186/s12885-016-2732-0 (PMC5002190; doi:10.1186/s12885-016-2732-0)
Supplement: Additional file 2: — Sensitivity analysis of forest plots included in our meta-analysis. (PDF 43 kb) [file 12885_2016_2732_MOESM2_ESM.pdf]

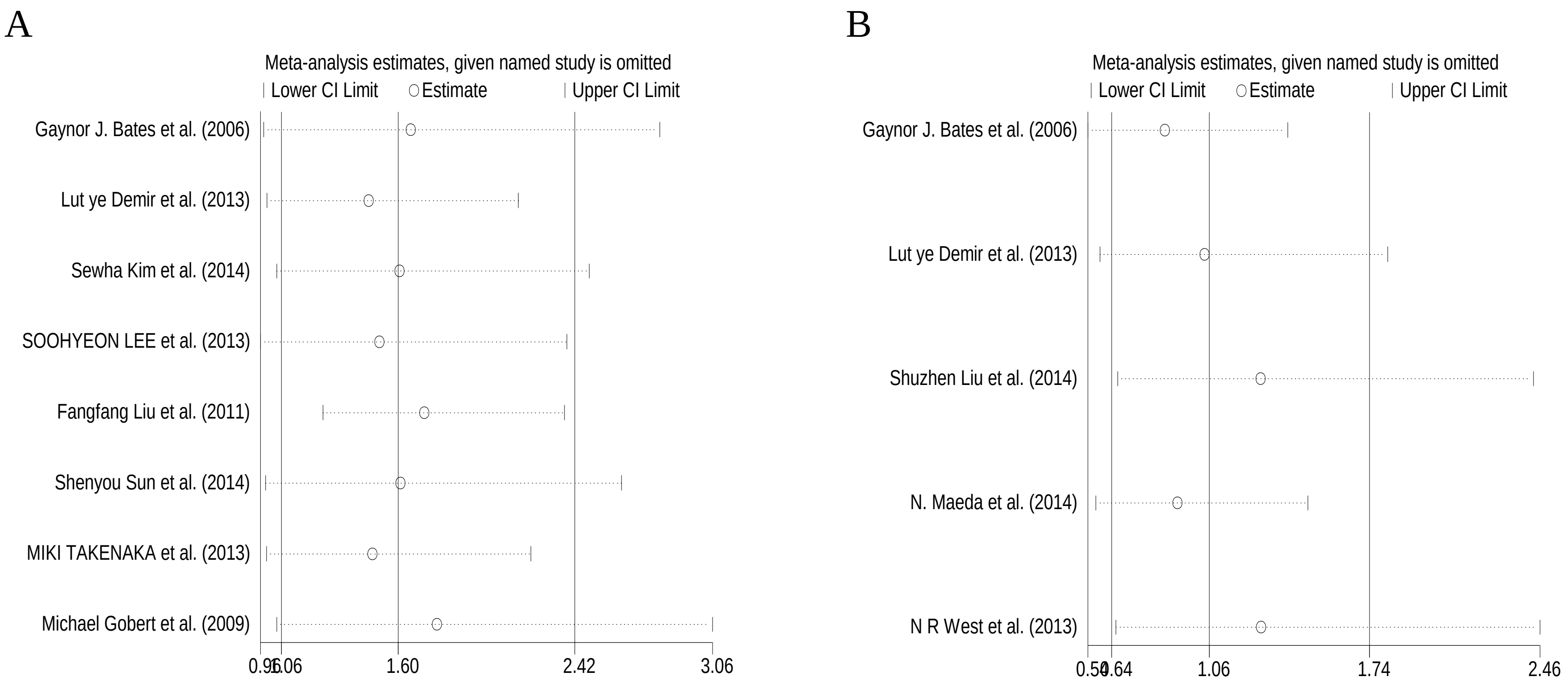

Figure S1: Sensitivity analysis of OS(A) and RFS(B).

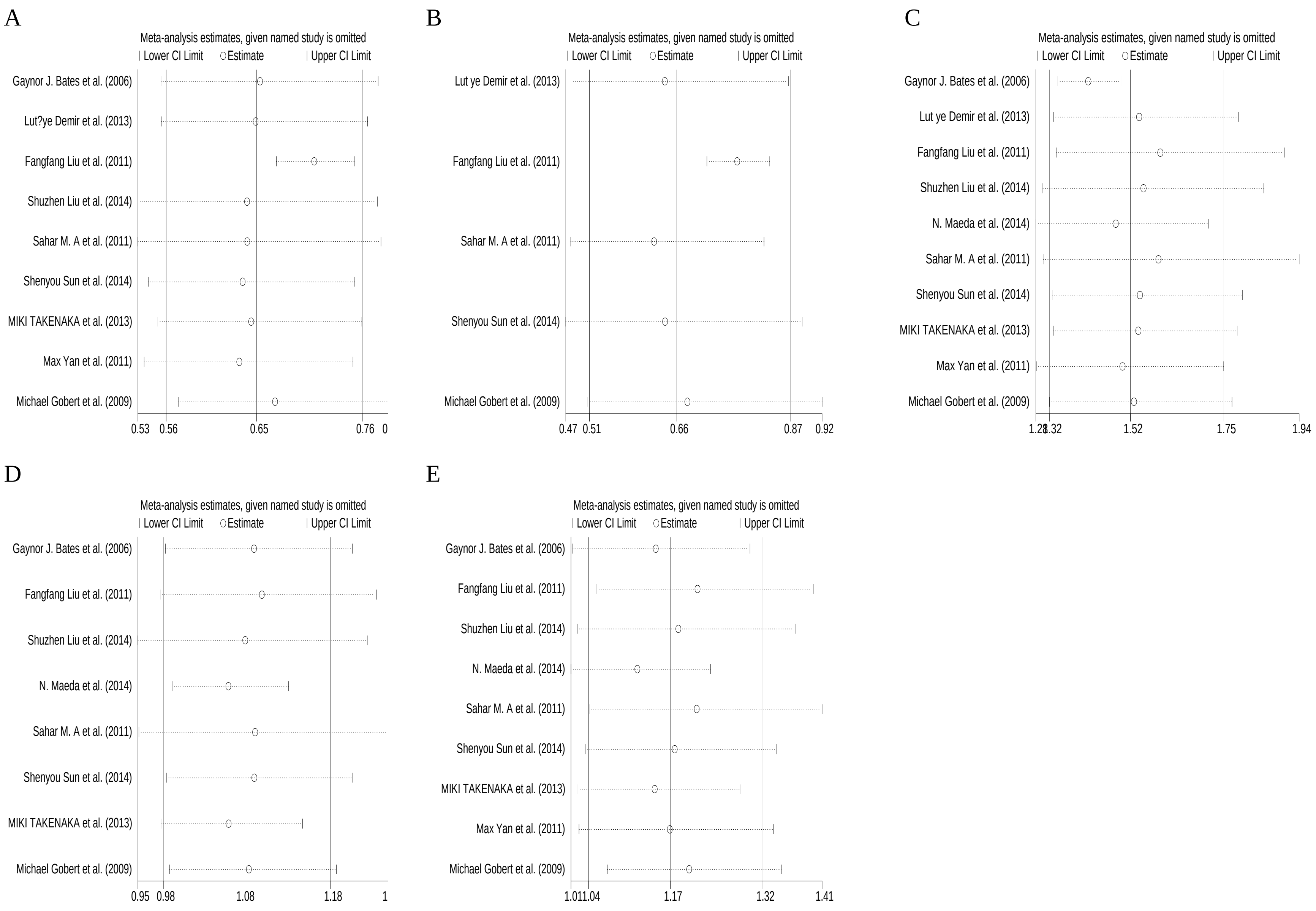

Figure S2: Sensitivity analysis of ER status(A) , PR status(B), c-erB-2 status(C), T category(D) and N category(E).
